# Exercise attenuates obesity-related cognitive and sleep-circadian dysfunctions by attenuating neuroinflammation via JAK/STAT in sex and age specific manner

**DOI:** 10.1038/s41366-026-02122-2

**Published:** 2026-06-02

**Authors:** Archana Yadav, Morgan Barkley, John C. Watson, Dev Patel, Andrew M. Pickering, Girish C. Melkani

**Affiliations:** 1 Department of Pathology, Division of Molecular and Cellular Pathology, Heersink School of Medicine, University of Alabama at Birmingham, Birmingham, AL, USA.; 2 Department of Integrative Biology and Pharmacology, University of Texas Health Science Center at Houston, Houston, TX, USA.; 3 These authors contributed equally: Archana Yadav, Morgan Barkley.

## Abstract

**BACKGROUND:**

Obesity including genetic obesity accelerates age-related cognitive decline and disrupts circadian regulation, increasing the risk of neurodegenerative disorders. These effects are mediated in part by metabolic dysregulation, neuroinflammation, and altered sleep–circadian cycles, yet the molecular and lifestyle factors that modify obesity-associated brain dysfunction remain poorly defined.

**OBJECTIVES:**

We investigated whether exercise mitigates metabolic, cognitive, and circadian dysfunction associated with genetic obesity, and whether exercise responses differ by sex and age.

**METHODS:**

Using *Drosophila melanogaster* (fruit fly), we compared Canton-S (CS) controls with flies carrying a loss-of-function mutation in sphingosine kinase 2 (*Sk2*), a conserved regulator of sphingolipid metabolism linked to obesity. Male and female flies were assessed at 3 and 6 weeks of age under exercised and non-exercised conditions. Outcomes included brain and peripheral lipid accumulation, synaptic marker expression, neuroinflammatory and apoptotic gene expression, sleep–circadian architecture, and olfactory aversive learning.

**RESULTS:**

Flies with *Sk2*-associated obesity exhibited increased lipid accumulation, synaptic dysregulation, neuroinflammation, sleep fragmentation, and cognitive impairment, with severity increasing with age. Endurance exercise significantly attenuated these abnormalities, but responses were sex- and age-dependent. Exercise reduced brain lipid burden and synapsin expression predominantly in males, whereas females showed greater suppression of inflammatory and apoptotic signaling. Sleep continuity and circadian stability improved markedly with exercise in females, while cognitive performance was rescued in both sexes, with stronger effects at younger ages.

**CONCLUSIONS:**

Endurance exercise mitigates obesity-associated metabolic, inflammatory, circadian, and cognitive dysfunction in a genetic *Drosophila* model, with distinct sex- and age-specific patterns of benefit. These findings highlight exercise as a powerful, non-pharmacological modifier of obesity-related brain aging/inflammation and underscore the importance of considering biological sex and intervention timing when designing strategies to promote healthy aging.

## INTRODUCTION

Obesity is a major global health challenge that accelerates age-related decline in brain function. Among the most harmful effects are cognitive decline and circadian rhythm disruption, which raise the risk of neurodegenerative diseases and lower quality of life in older adults [1]. These changes not only compromise individual well-being but also impose a significant societal burden [2]. The prevalence of obesity is expected to rise sharply; by 2035, more than half of the world’s population is expected to have obesity, potentially affecting over 4 billion people, which is like the COVID-19 pandemic. In 2022, ~16% of adults were living with obesity, and the global prevalence of adult obesity has more than doubled since 1990 [3, 4].

This growing epidemic is accompanied by increased obesity-related morbidity. Excess adiposity significantly elevates of type 2 diabetes, cardiovascular disease, non-alcoholic fatty liver disease, and several types of cancer [5–7]. These illnesses are caused by endothelial dysfunction, insulin resistance, dysregulated adipokine signaling, and chronic inflammation [5–7]. Collectively, these pathological alterations worsen the decline in brain health by causing systemic metabolic imbalance [5–7]. Genome-wide association studies (GWAS) have identified many single-nucleotide polymorphisms (SNPs) associated with obesity-related traits, highlighting a strong genetic link between obesity susceptibility, sleep/wake cycle regulation, and metabolic control [8]. Despite these insights, the mechanistic basis by which obesity interacts with aging to influence brain function remains incompletely understood.

Circadian rhythm disruption is a critical but underexplored consequence of obesity and aging. The circadian system orchestrates daily cycles of sleep, feeding, and hormonal regulation, maintaining metabolic and cognitive homeostasis. Obesity-induced alterations in circadian signaling impair sleep quality, disrupt neuronal plasticity, and exacerbate neuroinflammation, creating a vicious cycle that accelerates cognitive decline [9]. Importantly, memory processes are tightly regulated by circadian rhythms; circadian misalignment adversely affects hippocampal synaptic plasticity and impairs learning and memory performance [10–12]. Population-based studies have also demonstrated that insufficient sleep or poor sleep quality is associated with a heightened risk of developing metabolic and neurological disorders [13, 14]. Studies in humans and model organisms reveal that circadian misalignment increases vulnerability to metabolic disorders and neurodegenerative conditions, yet the molecular pathways linking these processes remain poorly defined.

One emerging mechanism underlying obesity-associated dysfunction involves sphingolipid metabolism, a critical regulator of cellular signaling and energy balance. The *Drosophila melanogaster Sk2* gene encodes sphingosine kinase 2, which converts sphingosine to sphingosine-1-phosphate (S1P), a bioactive lipid, that regulates metabolic and signaling pathways. Mutations like *Sk2*^*KG050894*^ lead to obesity-like phenotypes, including increased fat accumulation and hyperphagia [15, 16]. Recent studies further demonstrate that *Sk2* mutants also exhibit muscle dysfunction and metabolic impairment, which can be rescued by time-restricted feeding (TRF), highlighting the interplay between sphingolipid signaling, circadian rhythms, and energy homeostasis [15, 17]. The human ortholog, sphingosine kinase 2 (SPHK2), similarly regulates S1P levels, and is expressed in both adipose tissues and brain. Functional studies in mammals show that *Sphk2* knockout mice are protected against age-related obesity and insulin resistance, while pharmacological inhibition of SPHK2 reduces neuroinflammation in diet-induced obesity models [18, 19]. In addition, GWAS and expression quantitative trait loci (eQTL) analyses have linked SPHK2 to obesity, type 2 diabetes, and metabolic dysfunction [20] (https://www.gtexportal.org/home/gene/SPHK2). These findings, combined with adipose eQTL colocalization frameworks, suggest SPHK2 as a mechanistic contributor to cardiometabolic traits even when single-locus GWAS signals are modest. The conserved enzymatic function between *Sk2* and SPHK2 highlights a shared evolutionary mechanism in lipid signaling, making both genes valuable models for studying obesity-related disorders and potential therapeutic interventions.

Moreover, memory loss and dementia, especially among middle-aged individuals, have been strongly associated with obesity, however, there is no currently robust model for unraveling the mechanistic basis of this linkage [21–23]. Obesity-driven metabolic dysfunction is further associated with chronic low-grade inflammation, which disrupts systemic homeostasis and may contribute to cognitive decline and dementia [24, 25]. Inflammatory signaling in adipose tissue can impair insulin sensitivity, alter lipid metabolism, and promote neuroinflammation, yet the precise pathways connecting these processes to cognitive impairment remain elusive. Neuroinflammation, characterized by microglial activation and cytokine release, is increasingly recognized as a driver of synaptic dysfunction and memory loss in aging brain [26]. These processes may be amplified by obesity, creating a synergistic effect that accelerates neurodegeneration.

Lifestyle interventions such as exercise offer a promising strategy to counteract these detrimental effects. Exercise is widely recognized as a potent modulator of aging trajectories, improving metabolic health, reducing inflammation, and enhancing cognitive resilience in humans and model organisms [27, 28]. In *Drosophila*, endurance exercise has been shown to improve cardiac performance, climbing ability, and stress resistance, paralleling mammalian adaptations [29]. Exercise also influences mitochondrial biogenesis, lipid metabolism, and neuronal signaling, mechanisms that may mitigate obesity-induced dysfunction [30]. However, exercise responses are not uniform: they vary by sex, age, and genetic background. Males often exhibit stronger training adaptations than females, partly due to differences in octopaminergic signaling and energy metabolism [31]. Age further modulates these effects, with younger flies displaying greater exercise-induced benefits compared to older cohorts [32, 33]. Despite these observations, few studies have systematically examined how exercise interacts with sex, age, and genetic predisposition in the context of obesity and neurodegeneration.

*Drosophila melanogaster* provides an ideal model for addressing these questions. Its short lifespan, well-characterized genetics, and conserved metabolic pathways make it a powerful system for aging research [34, 35]. The availability of exercise paradigms such as rotational or negative geotaxis-based training enables controlled studies of endurance exercise in flies, allowing researchers to dissect molecular and physiological adaptations with precision. Moreover, sex-specific, and age-dependent differences in exercise response can be readily assessed, offering insights into biological variability that are difficult to capture in mammalian models.

Here, we address this critical gap by investigating the impact of endurance exercise on metabolic and functional traits in male and female *Drosophila* across two age groups (3 and 6 weeks), comparing Canton-S controls to *Sk2* mutants. Specifically, we aim to determine whether exercise mitigates obesity-like phenotypes in *Sk2* mutants, how sex and age influence susceptibility to exercise benefits, and which sex is more vulnerable to metabolic and functional decline without exercise. By integrating genetic, behavioral, and physiological analyses, our study provides novel insights into the interplay between lifestyle interventions, genetic risk, and biological sex in shaping aging trajectories. These findings have broad implications for understanding obesity-related cognitive decline and identifying targeted strategies to promote healthy aging.

## MATERIALS AND METHODS

### *Drosophila* models and diets

Canton-S and *Sphingosine kinase 2* (*Sk2* BDSC: 14133) were obtained from Bloomington *Drosophila* Stock Center (BDSC) and maintained at 25 °C. Canton-S (CS) was used as the wild-type control strain, as the Sk2 loss-of-function allele has been previously characterized and analyzed in a CS genetic background in multiple studies, where CS serves as the appropriate reference control [15, 17, 36]. Importantly, prior work demonstrated that *Sk2* heterozygous (Sk2/+) flies exhibit distinct muscle and metabolic phenotypes compared with both CS controls and *Sk2* homozygous mutants, indicating partial phenotypic penetrance rather than a true wild-type state. Accordingly, CS flies were selected as the most appropriate genetic background control for assessing Sk2-associated phenotypes. *Sk2* is a *Drosophila* ortholog of human sphingosine kinase, and *Sk2* loss-of-function mutants have been used in previous obesity-related studies in *Drosophila*, where they exhibit ceramide accumulation and obesity-like metabolic dysfunction [15, 17, 36]. Flies were maintained on a standard regular diet consisting of agar (11 g/L), active dry yeast (30 g/L), yellow cornmeal (55 g/L), molasses (72 mL/L), 10% nipagen (8 mL/L), and propionic acid (6 mL/L). Flies were housed at 25 °C and 50% humidity under a 12-h light/12-h dark (LD) cycle and transferred to fresh food every 3–4 days.

### Exercise regimens

Flies were subjected to a negative geotaxis-based exercise paradigm using a motorized rotational exercise apparatus, as previously described and validated [37]. Groups of flies housed in standard vials were placed into the device, which gently tapped the vials at 10-second intervals to induce repeated climbing behavior against gravity. This paradigm leverages the innate negative geotaxis response of *Drosophila* and provides a reproducible, non-invasive endurance exercise stimulus. Each daily training session lasted ~15 min and consisted of multiple cycles of induced climbing followed by brief rest periods, preventing exhaustion and minimizing stress-induced injury. Exercise was initiated early in adulthood, beginning at 1 week post-eclosion, to model early-life lifestyle intervention. For the 3-week cohort, flies were exercised daily from week 1 until 3 weeks of age, whereas for the 6-week cohort, flies were exercised daily from week 1 until 6 weeks of age. All exercise sessions were conducted at the same time each day to minimize circadian variability. Control (non-exercise) flies were handled identically, including placement near the apparatus during training sessions, but were not subjected to vial tapping or enforced climbing. Flies were maintained on standard diet conditions throughout the exercise period, and food vials were changed every 3–4 days to avoid confounding nutritional effects. This exercise protocol provides a moderate, non-lethal endurance stimulus that has been shown to improve metabolic, neuromuscular, and behavioral outcomes in *Drosophila* without reducing survival, and is widely used to investigate the interaction between physical activity, aging, and metabolic dysfunction [37].

### Olfaction aversion training assay

Flies were trained using the olfactory aversive conditioning method as outlined previously [38–40] and recently established in the lab [41]. Briefly, flies were exposed to two neutral odors, 3-octanol and 4-methylcyclohexanol, diluted to 1/10 in mineral oil. A 90–100 V, 60 Hz electrical shock was used as a reinforcer. The conditioning was conducted in a T-maze apparatus (CelExplorer Labs). The olfactory aversive conditioning procedure included three phases: naïve, training, and testing. In the naïve phase, flies were exposed to both odors for 3 min and 30 s to assess their odor preference based on the chamber in which the odor was presented. During a single training round, flies were exposed to one odor paired with the electrical shock for 2 min and 30 s, followed by 2 min and 30 s of exposure to the other odor without shock. The electrical shock was paired with the fly’s preferred odor, as determined in the naïve phase. After three rounds of training, flies were given a 10-min recovery period. In the testing phase, flies were given 3 min and 30 s to choose between the two odor chambers. After this time, the T-maze chambers were sealed, and the number of flies in each chamber was recorded. A performance index (PI) was calculated based on the flies’ avoidance of the odor associated with the shock. To validate these results, a sensory acuity test was performed using avoidance index calculations as previously described.

### Histology analysis

To evaluate the effects of exercise on CS and *Sk2* flies, histology was conducted as previously described [42–44]. Flies, exposed to the exercise regimen, were dissected and fixed in 4% paraformaldehyde (PFA) in phosphate-buffered saline (PBS) for 15 min at room temperature with gentle agitation. Following fixation, samples were rinsed three times for 10 min in PBS. The tissue was then equilibrated in a 15% sucrose solution in PBS before being embedded into molds containing optimal cutting temperature (OCT) compound. After freezing, samples were sectioned at 20 μm thickness using a Leica CM3050 S cryostat and mounted on glass slides (Fisher #15-188-48). Slides were allowed to air-dry for 30 min, washed briefly with PBS and blocked with 3% BSA prepared in TBS for 1 h. The slides were then incubated overnight at 4 °C with an anti-synapsin primary antibody (1:250, UI Developmental Studies Hybridoma Bank #3C11). After washing, slides were treated with AlexaFluor-750 anti-mouse fluorescent secondary antibody (1:500, Thermo Fisher # A-21037) together with the lipid marker LipidSpot 488 (1:100, Biotinum #70065) for visualization of lipid droplets. After washing, slides were mounted with ProLong Diamond Antifade Mountant containing DAPI (0.9 μg/ml, VECTA-SHIELD Vibrance H-1800). Fluorescence images were captured using an Olympus BX63F fluorescence microscope and analyzed with CellSens software at 10× magnification. DAPI fluorescence was used to define regions of interest (ROIs), as it clearly delineates brain substructure [14].

For quantitative analysis, lipid droplets (lipid objects) were defined as discrete LipidSpot-positive punctate structures following background subtraction and uniform thresholding. Lipid object count represents the number of individual lipid droplets per ROI, whereas lipid object area represents the mean cross-sectional area (μm^2^) of individual droplets, reflecting droplet size rather than total lipid content. Automated particle analysis was performed using the 488-nm channel with a minimum detection size of 20 pixels to exclude background signal. This approach to lipid droplet quantification, including analysis of droplet number and size, has been previously validated and employed in our prior studies examining lipid dysregulation in *Drosophila* models of metabolic and neurodegenerative disorders [42]. Fluorescence intensity measurements were quantified independently as mean fluorescence intensity within defined ROIs and were not used to derive lipid object number or area. Synaptic integrity was assessed by quantifying synapsin immunofluorescence intensity using a constant threshold in the 750-nm channel across all samples to ensure comparability between genotypes and treatment conditions. Synapsin immunofluorescence has been widely used as a reliable proxy for synaptic remodeling and neurodegeneration in *Drosophila* in our prior studies [17, 44].

All images were processed in batch mode using identical analysis parameters. For each condition, a minimum of three sections per fly and at least three flies per group were analyzed to ensure representative sampling of head and brain regions. Corresponding figure legends explicitly indicate whether lipid object count, lipid object area, or fluorescence intensity is represented in each graph.

### Sleep/circadian activity

We examined the sleep activity and fragmentation patterns of *Drosophila* under a 12-h light, 12-h dark (LD) cycle. Individual flies were monitored using the *Drosophila* Activity Monitoring System (DAM, TriKinetics Inc MA, USA) [41, 45]. We used 3-week-old male and female progeny of CS and *Sk2* with or without exercise. For analysis, we employed a machine learning-based app developed to seamlessly process the DAM data, enabling accurate measurements of sleep bouts, bout lengths, and frequency. *Drosophila* sleep was defined by a period of at least 5 min of inactivity, demonstrated by zero beam breaks recorded. Average sleep per 24 h (Zeitgeber Time; ZT) is a standardized way of measuring time within a circadian cycle, where ZT0 represents the beginning of the light phase and ZT12 marks the start of the dark phase), of each genotype, was calculated [41, 45]. Five days were used for analysis of 3-week-old flies.

### Real-time quantitative PCR

The heads of 3-week-old flies were carefully isolated and promptly frozen [41, 42, 45]. RNA was extracted using the Zymo Research Quick-RNA Microprep Kit, which included an on-column DNase I digestion step to ensure RNA purity. Quantitative PCR was carried out with the Sso Advanced Universal SYBR Green Supermix from Bio-Rad, utilizing the BIO-RAD CFX Opus Real-Time PCR System. Gene expression levels were normalized to the 60S ribosomal protein (Rpl11) as the reference. Three biological replicates were performed, each containing 8–10 flies. Primers for qPCR are listed below:

upd1-F: CAGCGCACGTGAAATAGCAT; upd1-R: CGAGTCCTGAGGTAAGGG GA;
upd3-F: ATCCCCTGAAGCACCTACAGA; upd3-R: CAGTCCAGATGCGTACTG CTG;
hop-F: CACCACCAACACCAATTC; hop-R: GGAACGTCGTTTGGCCTTCT;
Stat92E-F: CCTCGGTATGGTCACACCC; Stat92E-R: TGCCAAACTCATTGAG GGACT;
eiger-F: GATGGTCTGGATTCCATTGC; eiger-R: TAGTCTGCGCCAACATCATC;
hid-F: CACCGACCAAGTGCTATACG; hid-R: GGCGGATACTGGAAGATTTGC;
Rpl11-F: CGATCTGGGCATCAAGTACGA; Rpl11-R: TTGCGCTTCCTGTGGTTC AC;

Results are presented as 2^−ΔΔCt^ values normalized to the expression of Rpl11 and control samples. All reactions were performed in triplicate.

### Statistical analysis

Significance in sleep activity and qPCR gene analysis was determined using Two-way ANOVA with multiple comparisons Tukey’s test. Lipid droplets count and area differences were performed by two-way ANOVA with multiple comparisons Tukey’s test. Bar graphs show mean ± SEM. All statistical analyses were performed with Graph Pad Prism 10 and specific statistical test described under each figure legend.

## RESULTS

### Exercise counteracts obesity-linked lipid and synaptic dysregulation in a sex- and age-dependent manner

**Fig. 1:**
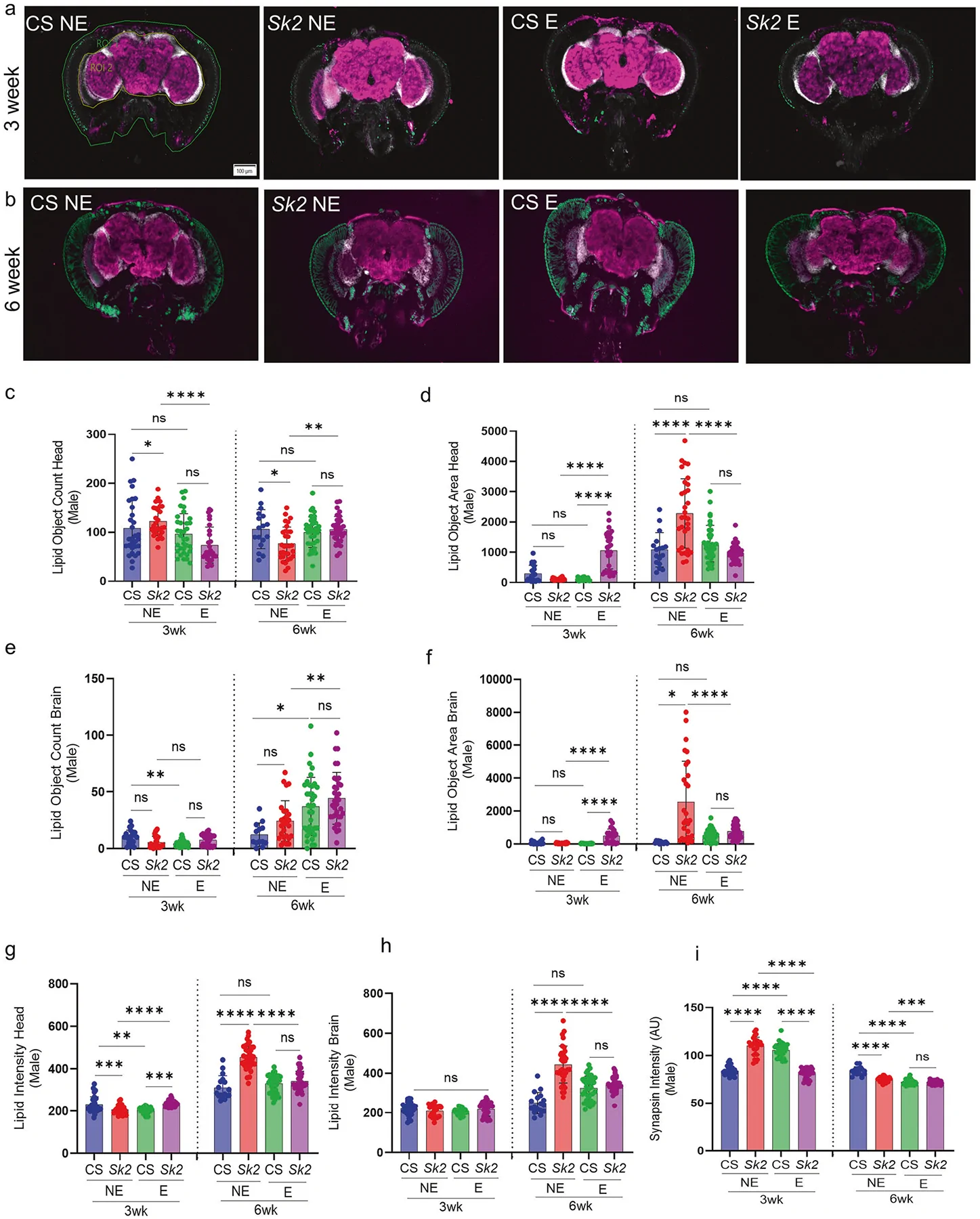
Effects of exercise on brain morphology and synaptic markers in male CS and *Sk2* groups at 3 and 6 weeks. Representative immunofluorescence images (**a**, **b**) illustrate brain sections from CS and *Sk2* groups under non-exercise (NE) and exercise (E) conditions at 3 weeks (**a**) and 6 weeks (**b**) in males. Green fluorescense represents lipid droplets (LipidSpot), magenta represents synapsin immunofluorescence (anti-SYNORF1), and nuclei are labeled with DAPI (white). Exercise significantly reduced obesity-associated lipid accumulation in the brains and head regions in male flies (**c**–**f**). In *Sk2* flies, lipid intensity increased in the head with no change in the brain at 3 weeks (**g**), whereas at 6 weeks it decreased in both regions (**h**). Synapsin intensity (**i**) was elevated in *Sk2* mutants compared to controls was reduced by exercise. All Images were acquired using identical exposure and acquisition settings across all genotypes and conditions. Data are representated as mean ± SEM with individual data points. Statistical analysis was performed using two-way ANOVA with Tukey’s multiple comparisons test (ns = *p* > 0.05, *p < 0.05, **p < 0.01, ***p < 0.0001). Quantification was performed from *n* = 5 flies/ genotype/ condition, with multiple sections analyzed per fly from ≥3 independent biological replicates. All the raw data including all the statistical significance are shown in the source file.

**Fig. 2:**
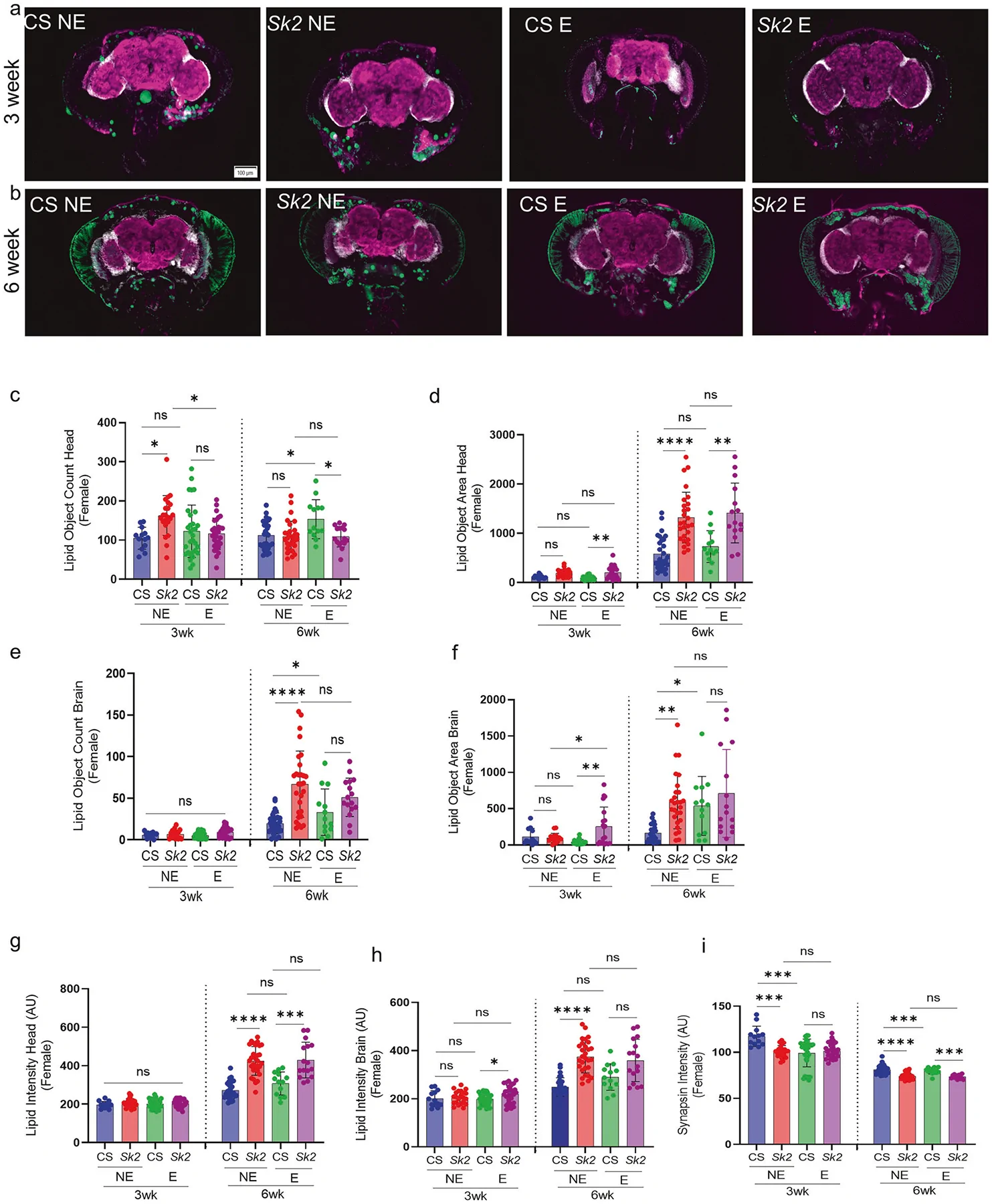
Effects of exercise on brain morphology and synaptic markers in female CS and *Sk2* groups at 3 and 6 weeks. Representative immunofluorescence images (**a**, **b**) show brain sections from CS and *Sk2* groups under non-exercise (NE) and exercise (E) conditions at 3 weeks (**a**) and 6 weeks (**b**) in females. Like males, green fluorescense represents lipid droplets (LipidSpot), magenta represents synapsin immunofluorescence (anti-SYNORF1), and nuclei are labeled with DAPI (white). At 3 weeks, exercise significantly reduced lipid object count in the brain (**c**, **d**), with no significant change in the head (**e**, **f**), or in lipid and synaptic intensity (**g**–**i**). By 6 weeks, exercise-driven effects were reduced, suggesting age-dependent responsiveness. All images were acquired under identical exposure settings. Data are presented as mean ± SEM with individual data points. Statistical analysis was performed using two-way ANOVA with Tukey’s multiple comparisons test (ns = *p* > 0.05, *p < 0.05, **p < 0.01, ***p < 0.0001). Quantification was performed from *n* = 5 flies/genotype/condition, with multiple sections analyzed per fly from ≥3 independent biological replicates. All the raw data including all the statistical significance are shown in the source file.

Immunofluorescence analysis revealed striking sex-, age, and region-specific differences in brain lipid distribution and synaptic marker expression under exercise (E) and non-exercise (NE) conditions (Figs. 1 and 2). Under NE condition, *Sk2* mutants exhibited significant lipid dysregulation compared to CS controls, with distinct differences between males and females, confirming obesity-linked metabolic and synaptic dysregulation [16]. These changes were evident in both 3 and 6 weeks, indicating that *Sk2* mutation accelerates lipid deposition and synaptic remodeling during aging.

In males, *Sk2* mutants displayed robust lipid accumulation in head, as evidenced by increased lipid object counts and intensity at 3 and 6 weeks (Fig. 1a–h). These changes were more pronounced in the head than brain (Fig. 1a–h), indicating heightened neuronal vulnerability to metabolic disruption. Exercise significantly mitigated these alterations, particularly, where lipid object counts and intensity were markedly reduced in *Sk2* mutants. Notably, exercise decreased lipid object area despite increased counts, suggesting a redistribution of lipid stores into fewer but larger droplets rather than complete clearance (Fig. 1c, d, g).

In contrast, females exhibited a distinct lipid profile. *Sk2* females showed elevated lipid object counts and intensity primarily in the head under NE conditions, while brain lipid parameters remained largely unchanged (Fig. 2a–h), indicating reduced neuronal susceptibility to lipid accumulation. Exercise had limited effects in females, with minimal changes in lipid object counts, area, or intensity in both head and brain, suggesting a lack of lipid remodeling in response to endurance training. Age further modulated these effects. At 6 weeks, lipid accumulation was exacerbated in *Sk2* mutants of females fly brain whereas reduced in male head with no change in brain. Exercise remained effective in reducing lipid object counts in older males (Fig. 1c, e), though improvements were smaller than at 3 weeks, consistent with age-related decline in exercise responsiveness [32, 33]. In females, exercise continued to primarily be reduced lipid object count in brain without reducing lipid object area, reinforcing persistent sex-specific differences in lipid remodeling with age (Fig. 2c–f).

Synaptic marker analysis further highlighted sex-dependent effects. In males, synapsin intensity was significantly elevated in *Sk2* mutant under NE conditions compared to CS controls, indicative of synaptic dysregulation associated with metabolic stress (Fig. 1i). Exercise reduced synapsin levels, suggesting partial restoration of synaptic homeostasis. In contrast, females exhibited no significant changes in synapsin expression under exercise conditions, indicating preserved synaptic stability despite lipid alternations (Fig. 2i). These findings align with previous reports that endurance exercise improves brain lipid metabolism and synaptic health in aging flies [46] and highlight sex-dependent differences in exercise benefits [31, 47].

Collectively, these results demonstrate that endurance exercise mitigates obesity-associated lipid and synaptic alterations in *Drosophila*, with distinct sex- and age-dependent patterns of brain remodeling. Males exhibit greater exercise-induced reductions in lipid counts and synapsin intensity, while females show structural remodeling of lipid deposits without major changes in synaptic markers, underscoring the complexity of sex-specific responses to metabolic stress and physical activity.

### Exercise modulates peripheral lipid and synaptic expression in flies with obesity

To assess whether these effects were brain-specific or reflected systemic responses, we examined lipid and structural markers in the thorax and abdomen. In *Sk2* mutant males, exercise significantly reduced elevated lipid object counts in both regions, with no change in area, suggesting modulation of lipid load without altering droplet size (Supplementary Fig. 1a–j). In females, lipid counts, and area were elevated in the thorax and reduced with exercise, while no changes were observed in the abdomen, indicating limited plasticity (Supplementary Fig. 2a–j). Lipid intensity in the thorax was decreased under exercised conditions in male (Supplementary Fig. 1e). Given synapsin’s presence in neuromuscular junctions and gut [48, 49], we also analyzed peripheral tissues. Synapsin was elevated in the thorax and abdomen of *Sk2* males but reduced by exercise only in the thorax, indicating region-specific normalization (Supplementary Fig. 1f, j). In females, synapsin in the thorax followed similar patterns, both elevated in *Sk2* mutants and reduced with exercise, while remaining unchanged in the abdomen at 3 weeks (Supplementary Fig. 2f, j). By 6 weeks, these exercise-driven effects were reduced or absent, reinforcing the notion of an early developmental window for exercise-induced plasticity. Sex comparisons revealed that females generally exhibited higher lipid intensity and synaptic marker levels in the abdomen under enriched conditions at 3 weeks compared to males, suggesting a greater responsiveness to exercise during early development. These observations are consistent with reports that sex hormones modulate lipid metabolism and synaptic plasticity, influencing responsiveness to environmental stimuli. Mechanistically, exercise may stimulate lipid metabolism and synaptic plasticity in peripheral regions through systemic metabolic signaling and neuromuscular activity, which enhance mitochondrial function and neuroplasticity.

### Exercise attenuates neuroinflammation and apoptotic signaling in *Sk2* mutants: sex- and age-dependent effects

**Fig. 3:**
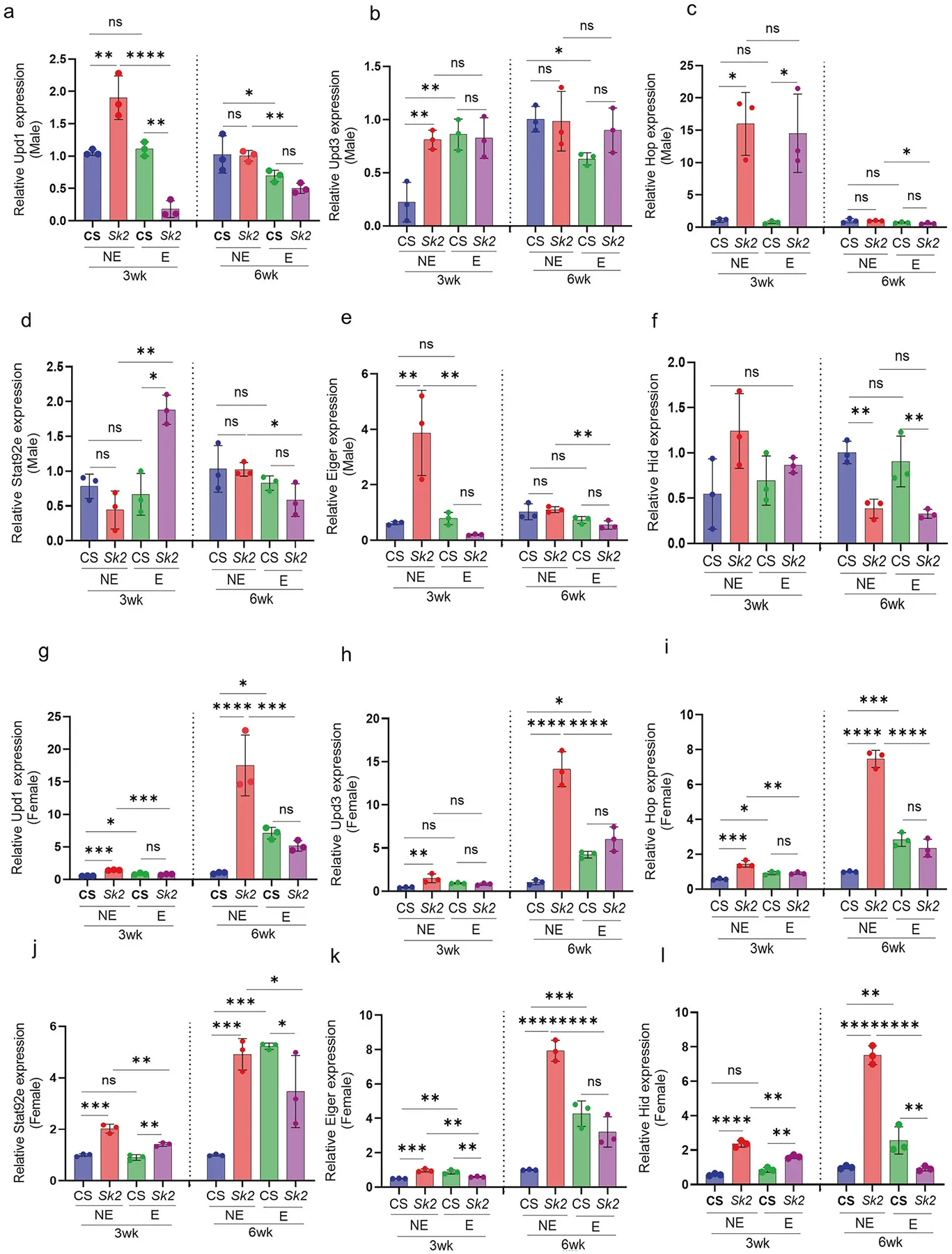
Impact of exercise on cytokine, signaling, and apoptosis-related gene expression in female and male CS and *Sk2* groups at 3 and 6 weeks. Quantitative PCR (qPCR) analysis of female brains (**a**–**f**) revealed expression of inflammatory cytokines (*upd1*, and *upd3*), JAK/STAT components (*hop, Stat92E*), along with the pro-inflammatory marker (*eiger*) in *Sk2* mutant at both 3 and 6 weeks. Exercise strongly reduced expression of these genes, particularly in females at 6 weeks. Expression of the pro-apoptotic genes *hid* was also reduced, indicating suppression of apoptotic signaling. In males (**g**–**l**), similar trends were observed, with elevated inflammatory and apoptotic gene expression in *Sk2* mutants and moderate reduction upon exercise. Exercise had minimal effects on control flies. Data are presented as mean ± SEM. Statistical analysis was performed using two-way ANOVA with Tukey’s multiple comparisons test (ns = *p* > 0.05, *p < 0.05, **p < 0.01, ***p < 0.0001). qPCR analysis was performed using *n* = 3 biological replicates, each consisting of pooled samples from 8 to 10 flies. All the raw data including all the statistical significance are shown in the source file.

Quantitative PCR (qPCR) analysis revealed that *Sk2* mutants exhibit a pronounced neuroinflammatory profile characterized by elevated expression of cytokines (*upd1*, and *upd3*) and JAK/STAT signaling components (*hop, Stat92E*), along with the pro-inflammatory marker *(eiger)* and pro-apoptotic gene *hid* compared to CS controls (Fig. 3a–l). These changes were evident at both 3 and 6 weeks, indicating that obesity accelerates inflammatory and apoptotic signaling in the brain [15, 16]. Notably, the magnitude of dysregulation was greater in females than males, particularly at 6 weeks, where *Sk2* NE females showed dramatic upregulation of *upd* genes and JAK/STAT components (Fig. 3a–f), suggesting heightened vulnerability to chronic inflammation with age [47].

Exercise significantly mitigated these effects in both sexes, but the response was stronger in females. In *Sk2* females, exercise reduced expression of *upd1* and *upd3* and JAK/STAT components by 50–70% at 6 weeks, while also lowering *eiger* and apoptotic markers (Fig. 3a–f). In males, exercise produced moderate reductions in *upd* genes and *eiger* at 3 weeks, with smaller effects at 6 weeks (Fig. 3g–l). These findings suggest that exercise exerts a neuroprotective effect by suppressing inflammatory cytokine signaling and apoptosis, with greater efficacy in females and during later life stages [46].

Mechanistically, the stronger female response may reflect sex-specific differences in immune regulation and metabolic stress adaptation, as previously reported in *Drosophila* models [31, 47]. The age-dependent attenuation of exercise benefits in males aligns with evidence that chronic obesity and aging impair stress resilience and signaling plasticity [32, 33]. Interestingly, the observed increase in *upd3* expression in thoracic tissue following exercise (Supplementary Fig. 3a–l) may indicate a JAK/STAT-mediated regenerative response linked to muscle remodeling and systemic metabolic adaptation [15].

### Exercise mitigates cognitive deficits in *Sk2* mutants

**Fig. 4:**
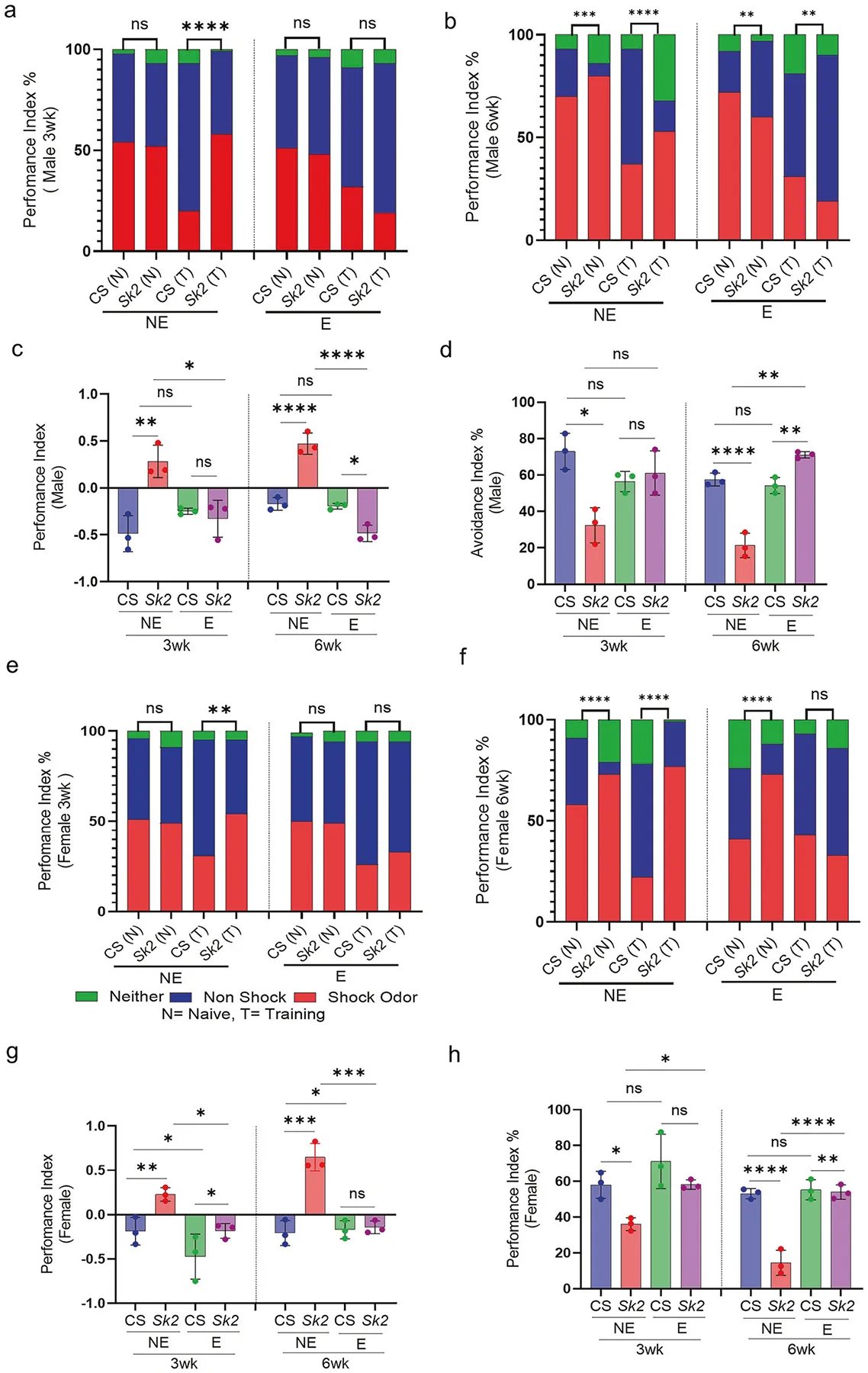
Consequences of exercise on cognitive performance and avoidance behavior in CS and *Sk2* groups at 3 and 6 weeks. Exercise rescued learning and memory deficits in *Sk2* mutants. Non-exercised *Sk2* males and females exhibited reduced performance indices at both 3, and 6 weeks of age (**a**, **b**, **e**, and **f**). Exercise improved memory retention. Decision making (**c**, **g**) and odor avoidance behavior (**d**, **h**) were impaired in *Sk2* mutants and restored with exercise. Data are presented as mean ± SEM with individual data points. Statistical analysis was performed using Chi-square tests (**a**, **b**, **e**, **f**)) and Two-way ANOVA with Tukey’s post-hoc (**c**, **d**, **g**, **h**). Data are shown as percentages of flies in each chamber at assay conclusion (*n* = 60–150 flies/genotype/condition). All the raw data including all the statistical significance are shown in the source file.

To assess cognitive dysfunction in flies with obesity, we evaluated olfactory aversion learning. Non-exercised *Sk2* mutants exhibited pronounced impairments in short-term learning and memory at both 3 and 6 weeks, as shown by reduced olfaction memory performance indices in aversive conditioning assays and decision-making tasks (Fig. 4a–h). These deficits were accompanied by odor avoidance impairments, indicating compromised cognitive performance (Fig. 4c, d, g, h). Exercise intervention significantly rescued these deficits, restoring memory retention and avoidance behavior to levels comparable to Canton-S (CS) controls. Improvements were observed in both sexes, but females demonstrated greater benefits at 6 weeks, particularly in odor avoidance and decision-making tasks (Fig. 4c, d, g, h). Rescued cognitive performance is likely mediated by exercise-induced enhancements in sleep architecture and circadian stability. Sleep plays a critical role in memory acquisition and consolidation in *Drosophila*, and sleep deprivation disrupts these [50, 51]. Exercise stabilizes circadian rhythms, increases total sleep, and reduces fragmentation in aging flies, which supports memory consolidation [46, 52]. Furthermore, circadian disruption impairs memory consolidation, highlighting the importance of rhythmicity for cognitive resilience [53]. Our findings align with these studies, suggesting that exercise mitigates obesity-driven cognitive decline by improving sleep–wake regulation and reducing circadian misalignment. This dual benefit, restored sleep continuity and memory performance, underscores the systemic role of exercise in promoting neurobehavioral health during aging. These findings suggest that obesity driven by *Sk2*-mediated metabolic dysfunction increases the risk of cognitive deficits, particularly in learning-memory tasks. This aligns with human studies linking midlife obesity to increased susceptibility to cognitive decline and neurodegenerative disorders [21, 23].

### Effects of obesity and exercise on sleep–circadian cycle

**Fig. 5:**
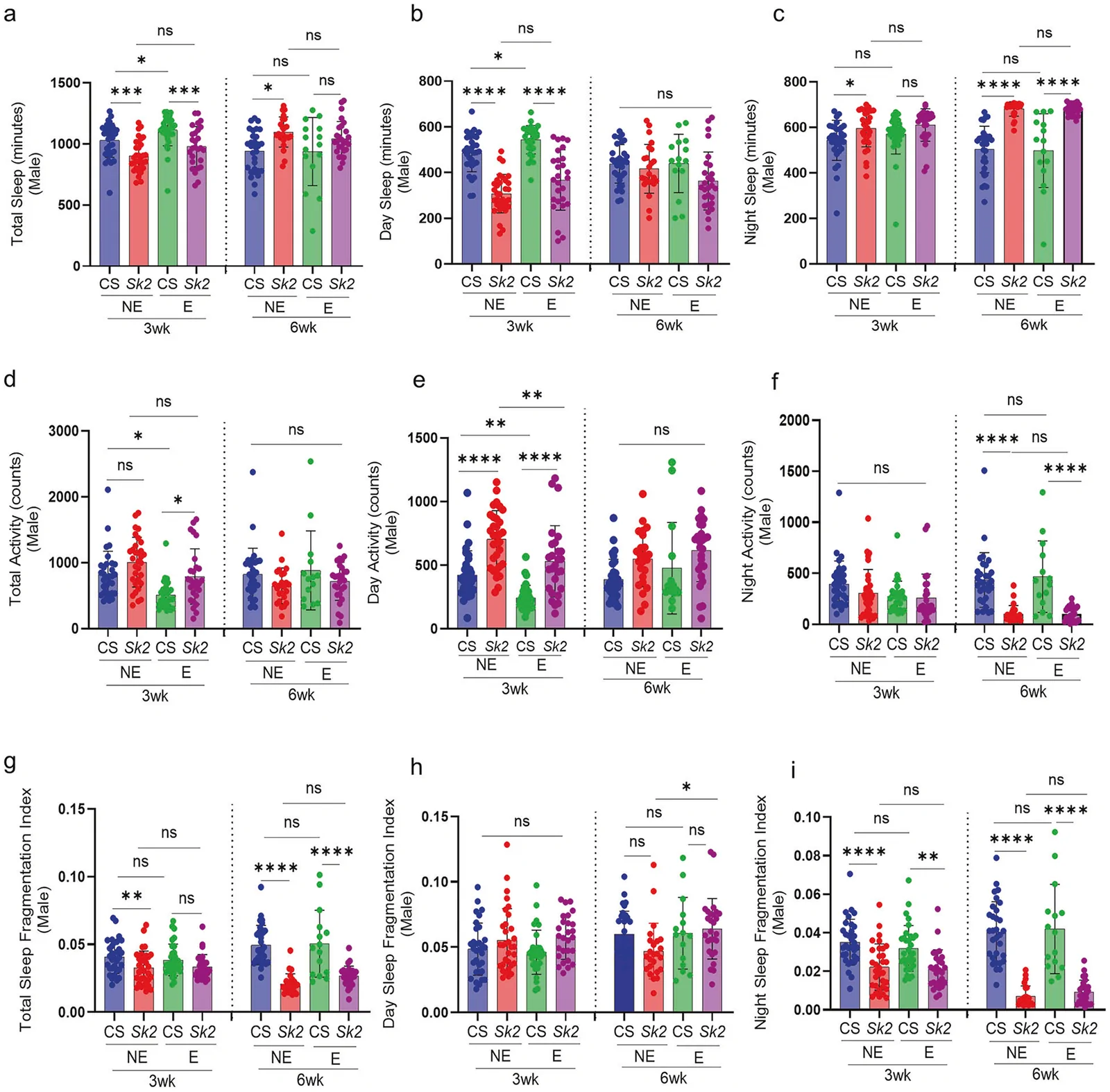
Impact of exercise on sleep architecture, and sleep fragmentation in CS and *Sk2* male *Drosophila* at 3 and 6 weeks. At 3 weeks, exercise increased total, and daytime time (**a**, **b**) with minimal change in night sleep (**c**). Activity levels are modestly reduced (**d**–**f**) and sleep fragmentation showed slight improvement (**g**–**i**). At 6 weeks, exercise effects were reduced, with minimal change in sleep duration and activity, although sleep fragmentation indices showed modest improvement during daytime. Data are presented as mean ± SEM. Statistical analysis was performed using Two-way ANOVA with Tukey’s multiple comparison test (*n* = 32 flies/group). ns = *p* > 0.05, *p < 0.05, **p < 0.01, ***p < 0.0001). All the raw data including all the statistical significance are shown in the source file.

To assess the impact of obesity and exercise on sleep architecture and circadian activity, we analyzed CS flies and *Sk2 m*utants, across age and sex [15, 36]. Sleep and activity patterns were monitored under 12-h light/12-h dark cycle using standard *Drosophila* activity monitoring protocols [15, 36]. 3week-old male *Sk2* mutant flies showed disrupted sleep and activity patterns compared to controls, with reduced total sleep, decreased daytime sleep, and increased nighttime sleep (Fig. 5a–c). Daytime activity was markedly elevated, indicating circadian rhythm disruption, and sleep architecture was fragmented, as shown by shorter bout lengths (Supplementary Fig. 5a–f). These findings align with previous reports linking obesity and circadian misalignment in flies [54].

Exercise produced modest improvements in 3 weeks. Exercised *Sk2* males showed partial restoration of daytime sleep compared to non-exercised (NE) groups (Fig. 5b), although night sleep remained unchanged. Activity counts decreased slightly in exercise groups, particularly during the day, while night activity was unaffected (Fig. 5d–f). Sleep fragmentation indices improved marginally, with exercised *Sk2* males exhibiting longer bout lengths than NE counterparts (Fig. 5g–i, Supplementary Fig. 4d–f), suggesting limited early-life exercise benefits.

**Fig. 6:**
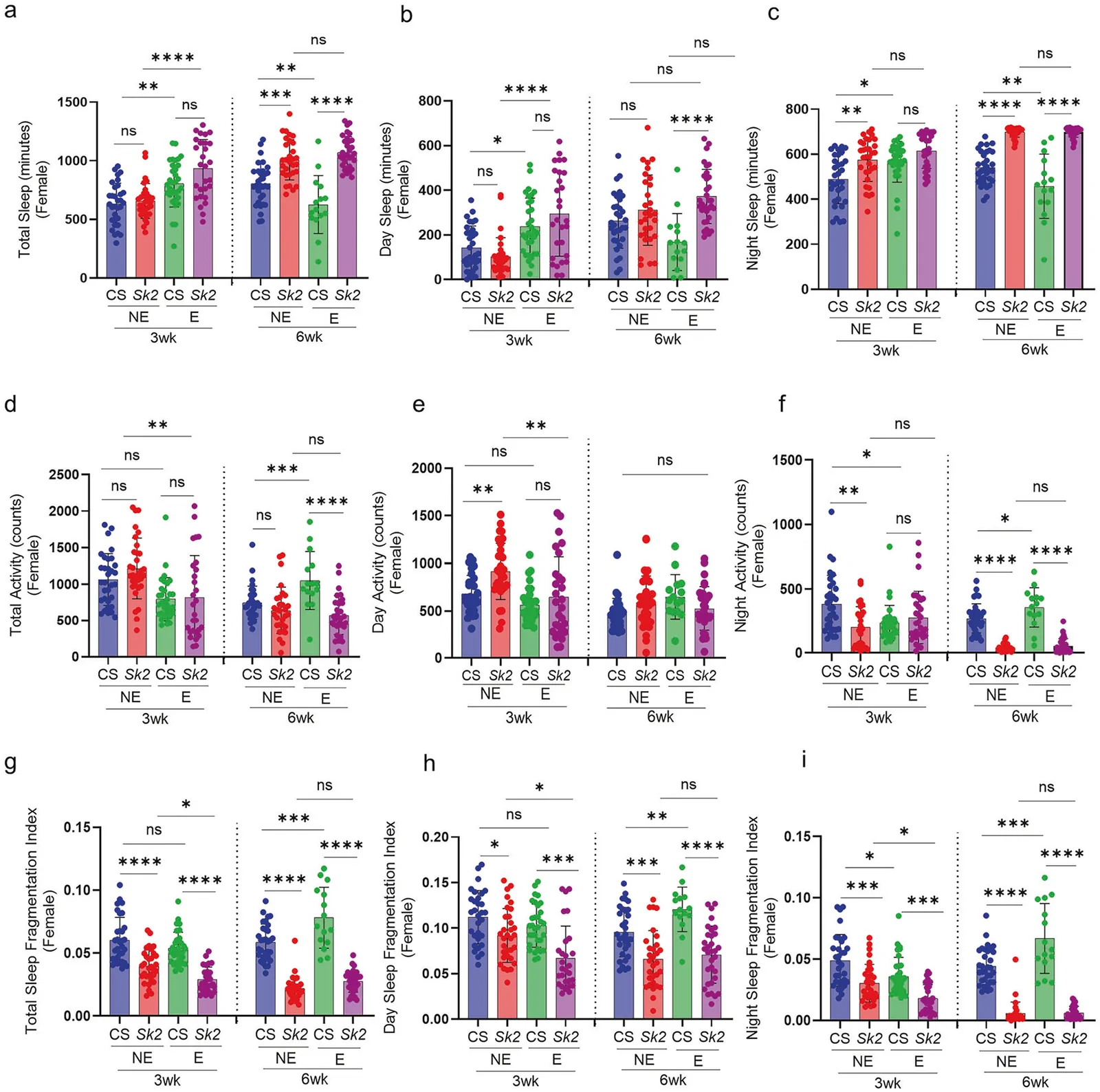
Impact of exercise on sleep architecture, and sleep fragmentation in CS and *Sk2* female Drosophila at 3 and 6 weeks. Exercise significantly increased total and daytime sleep in females (**a**, **b**), with additional improvements in night sleep (**c**). Activity levels decreased with exercise (**d**–**f**), and sleep fragmentation was significantly reduced, particularly in *Sk2* females at 6 weeks (**g**–**i**). These findings indicate improved sleep continuity and stability with exercise. Data are presented as mean ± SEM. Statistical analysis was performed using two-way ANOVA with Tukey’s multiple comparisons test (*n* = 32 flies per group). significance levels: ns = *p* > 0.05, *p < 0.05, **p < 0.01, ***p < 0.0001. All the raw data including all the statistical significance are shown in the source file.

By 6 weeks, exercise effects were diminished. Sleep duration (total, day, night) did not differ significantly between E and NE groups, and activity counts dropped only slightly, particularly night activity in Sk2-E males sleep (Fig. 5a–f). Fragmentation indices improved marginally in *Sk2*-E males during daytime, indicating some stabilization of sleep continuity with age (Fig. 5g–i). Overall, the graphs show that at 3 weeks, *Sk2-NE* males had significantly lower total and daytime sleep compared to CS-NE, while exercise partially restored daytime sleep. Bout length data reveal that *Sk2*-NE males exhibited shorter bouts at both ages, reflecting fragmented sleep, and exercise improved bout length modestly at 3 weeks but not at 6 weeks. Female *Sk2* mutants displayed similar circadian disruptions to males, with more sleep occurring at night than during the day, shorter sleep bouts, and increased fragmentation compared to CS controls (Fig. 6a–i, Supplementary Fig. 4g–l). Females showed stronger significant effect than males but exhibited comparable activity patterns, characterized by higher daytime activity and lower nighttime activity under NE conditions (Fig. 6a–f).

Exercise produced more pronounced benefits in females than in males. At 3 weeks, exercise significantly increased total sleep and daytime sleep in both CS and *Sk2* females, with the largest improvement observed in *Sk2* mutants. Night sleep also increased with exercise in CS and *Sk2*. Activity counts decreased in exercised females, particularly during the day, and night activity dropped sharply at 6 weeks, in *Sk2* females (Fig. 6a–f). Sleep fragmentation indices were significantly lower in exercised females compared to NE groups, with *Sk2* females showing the greatest reduction at 6 weeks (Fig. 6g–i), indicating improved sleep continuity. Under NE conditions, *Sk2* females exhibited higher fragmentation and lower activity than CS, but exercise mitigated these differences. These findings are consistent with previous reports that endurance exercise improves sleep–wake stability and reduces fragmentation in aging *Drosophila*, particularly in females [46, 52]. Moreover, sex-dependent differences in sleep architecture and circadian vulnerability have been documented, with females showing greater susceptibility to disruption but also benefiting from interventions [47, 55].

## DISCUSSION

This study demonstrates that exercise exerts broad neuroprotective effects in a genetic model of obesity, mitigating multiple pathological processes associated with aging and metabolic stress. Using *Sk2* mutants, which exhibit sphingolipid dysregulation and obesity-like traits [16], we observed profound disruptions in sleep architecture, cognitive performance, lipid metabolism, synaptic integrity, and inflammatory signaling. These findings align with evidence that obesity accelerates neurodegenerative risk through chronic inflammation, lipid accumulation, and circadian misalignment [15, 47].

Exercise significantly improved sleep continuity and reduce fragmentation, particularly in females, which is consistent with prior reports that physical activity stabilizes circadian rhythms and enhances sleep quality in aging flies [46, 52]. Improved sleep likely contributed to the observed rescue of learning and memory deficits, as sleep is essential for memory consolidation in *Drosophila* [50, 51]. These behavioral benefits underscore the interplay between metabolic health, circadian regulation, and cognitive resilience.

Interestingly, exercise exhibited sex-specific divergence in its effects on behavioral outcomes. While both sexes benefited from exercise, both genders show rescue male and in cognitive performance, whereas females exhibited more pronounced improvements in sleep parameters. These differences may reflect sex-specific neuromodulators and metabolic pathways. Previous studies suggest that octopaminergic signaling and metabolic responses to endurance exercise differ between males and females, potentially driving divergent physiological adaptations [31, 56]. Additionally, baseline metabolic differences associated with phenotype severity may affect responsiveness to intervention, with females showing greater sleep fragmentation, while males and females exhibiting makeable cognitive deficits. Therefore, it is vital to emphasize the importance of considering biological sex and intervention timing when designing strategies to promote healthy aging.

At the structural level, *Sk2* mutants displayed marked lipid deposition and elevated synapsin intensity in the brain, indicating obesity-driven metabolic stress and synaptic remodeling. Exercise reduced lipid object counts and normalized synapsin levels, suggesting improved lipid clearance and synaptic homeostasis. Interestingly, females exhibited structural remodeling of lipid deposits without major synapsin changes, whereas males showed stronger synaptic normalization, Exercise reduced lipid object. These sex-specific patterns may reflect differences in energy allocation and neuronal plasticity, as previously reported in endurance exercise studies [31]. Age further influenced these effects: while exercise remained beneficial at 6 weeks, the magnitude of improvements was reduced compared to younger cohorts, indicating an age-dependent decline in responsiveness. Molecular profiling revealed robust activation of inflammatory cytokines (*upd1*, *upd3*), JAK/STAT components (*hop*, *Stat92E*), and pro-apoptotic genes (*hid*) in *Sk2* brains, indicating a state of chronic neuroinflammation and cell death risk. Exercise markedly downregulated these pathways, particularly in females at 6 weeks, suggesting enhanced neuroprotection during later life stages [46]. The observed increase in *upd3* expression in thoracic tissue following exercise may represent a JAK/STAT-mediated regenerative response linked to muscle remodeling and systemic adaptation [15]. These findings align with mammalian studies showing that physical activity reduces neuroinflammatory signaling and preserves synaptic integrity during aging [57, 58].

**Fig. 7:**
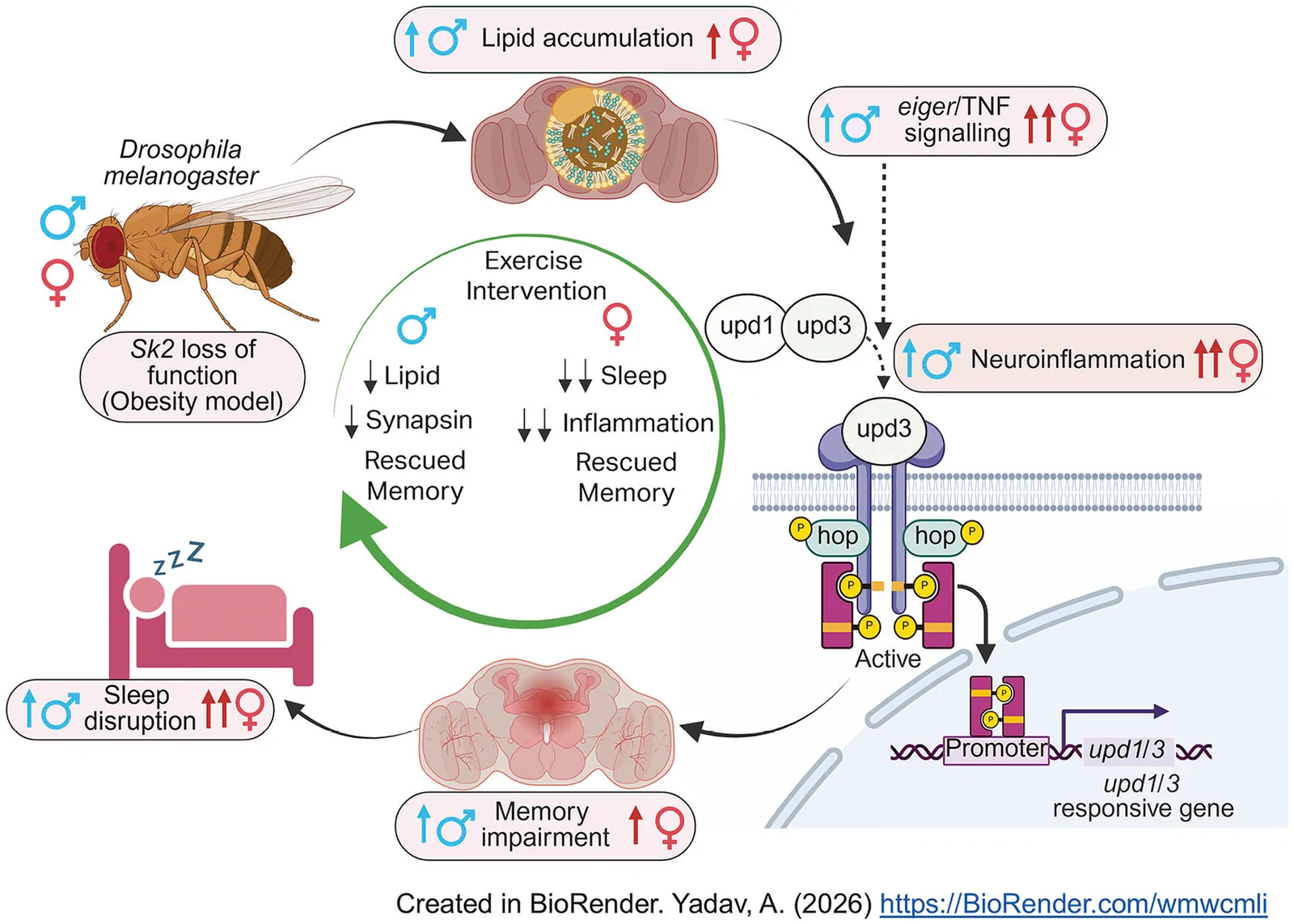
Schematic representation of sex-specific effects of exercise in *Sk2*-mediated obesity. The schematic illustrates loss of *Sk2* function induces an obesity-like phenotype characterized by increased lipid accumulation, activation of *eige*r/TNF signaling, and elevated neuroinflammation through *upd1*/*upd3*-mediated JAK/STAT pathway activation. These changes are associated with disrupted sleep–circadian architecture and impaired memory function in both males (♂) and females (♀). Exercise intervention differentially modulates these phenotypes in a sex-dependent manner. In males, exercise primarily reduces lipid accumulation and partially suppresses neuroinflammatory signaling, leading to improvements in memory and sleep. In females, exercise exerts stronger anti-inflammatory effects, resulting in significant improvements in sleep–circadian stability and cognitive function. Arrows indicate direction and magnitude of change (↑ increase, ↓ decrease, ↑↑ stronger increase, ↓↓ stronger reduction). Male (♂) and female (♀) symbols denote sex-specific responses. Image generated using Biorander.

Collectively, our results highlight sex and age as critical determinants of exercise benefit. Females exhibited greater improvements in sleep quality, inflammatory suppression, memory rescue and metabolic remodeling, whereas males showed stronger synaptic recovery, and memory rescue. These differences may arise from sex-specific hormonal and neuromodulators pathways influencing energy balance and immune regulation [31, 47]. Age consistently attenuated exercise benefits, emphasizing the importance of early-life interventions to maximize neuroprotective outcomes (Fig. 7).

These findings are consistent with mammalian studies demonstrating that exercise reduces neuroinflammation and improves cognitive function in metabolic disorders [59–61]. Exercise has been shown to enhance hippocampal plasticity, reduce inflammatory signaling, and improve memory performance. Given the conserved roles of JAK/STAT signaling and lipid metabolism across species [62], similar mechanisms may operate in humans, highlighting exercise as a potential non-pharmacological intervention for obesity-associated neurodegenerative conditions [63].

Given the evolutionary conservation of lipid metabolism, circadian regulation, and JAK/STAT signaling, these findings have translational relevance for designing lifestyle interventions to combat obesity-related cognitive decline and neurodegenerative risk in humans. Future studies should explore molecular mediators of sex-specific exercise responses and assess whether combining exercise with dietary or pharmacological strategies can further enhance neuroprotection during aging.

## Supplementary Material

SI Material

The online version contains supplementary material available at https://doi.org/10.1038/s41366-026-02122-2.

## Data Availability

The data that support the findings of this study are available as source data and can be also requested from the corresponding author.
