# Supplementary material for: Exercise attenuates obesity-related cognitive and sleep-circadian dysfunctions by attenuating neuroinflammation via JAK/STAT in sex and age specific manner
Source: Int J Obes (Lond). 2026 Jun 2;50(9):1953–66. doi: 10.1038/s41366-026-02122-2 (PMC13492066; doi:10.1038/s41366-026-02122-2)
Supplement: Supplementary file 1 [file 41366_2026_2122_MOESM1_ESM.pdf]

Exercise Mitigates Obesity-Related Cognitive and Circadian Dysfunctions by Attenuating Neuroinflammation via JAK/STAT

Archana Yadav[1#], Morgan Barkley[1#], John C. Watson[1], Dev Patel[1], Andrew M. Pickering[2], Girish C. Melkani[1*]

[1]Division of Molecular and Cellular Pathology, Department of Pathology, Heersink School of Medicine, University of Alabama at Birmingham, Birmingham, AL, USA.[2]Department of Integrative Biology and Pharmacology, University of Texas Health Science Center at Houston, Houston, TX, 77030, USA

Correspondence: *Girish C. Melkani, girishmelkani@uabmc.edu;

# Equal contribution.

Supplementary Information

Supplementary Figures 1-5

Supplementary Fig. 1

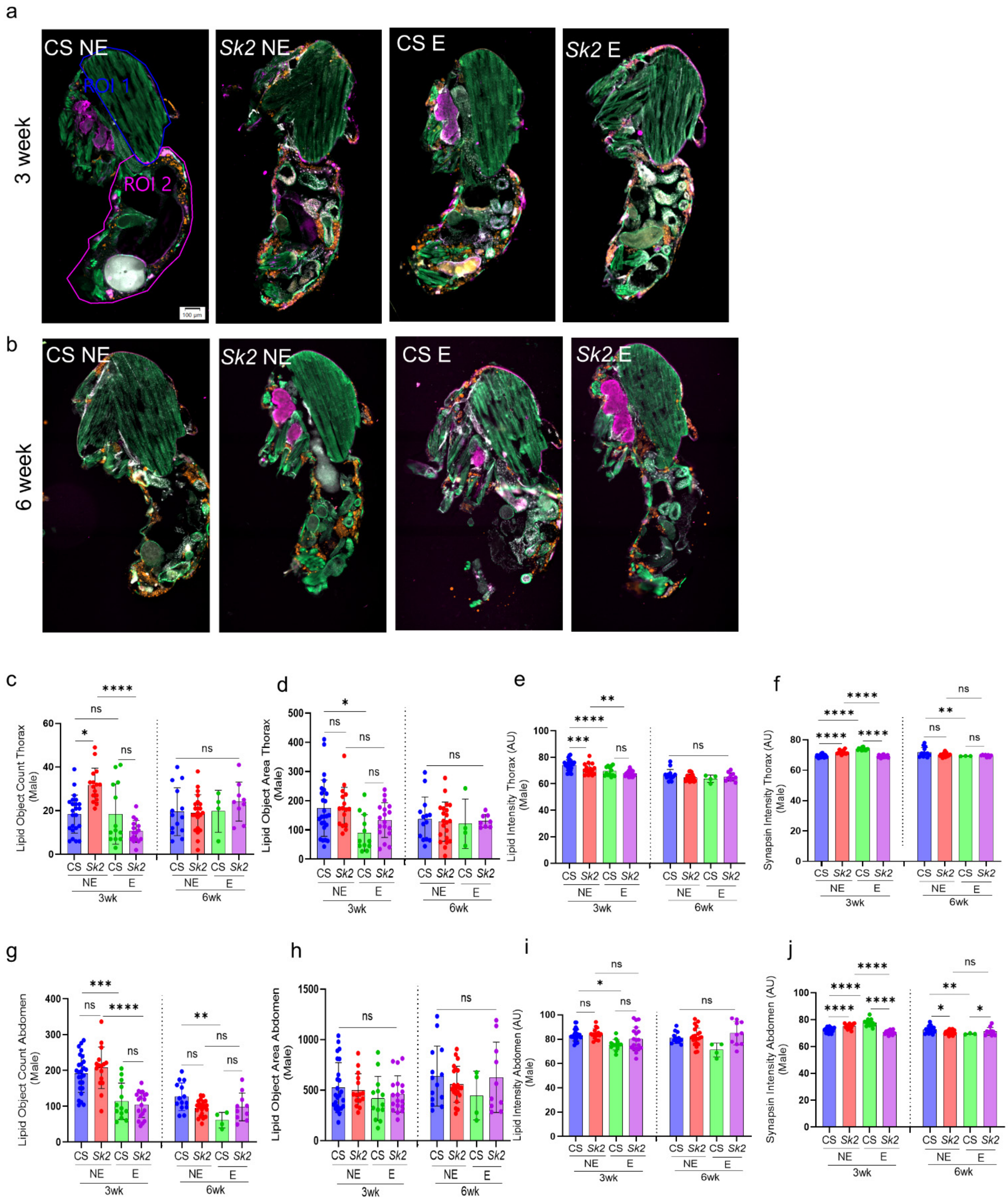

Supplementary Fig. 1. Effects of exercise on thorax and abdomen morphology in male CS and *Sk2* groups at 3 and 6 weeks.

(a, b) Representative images showing lipid accumulation (orange), phalloidin staining (green), synapsin (magenta, indicating synaptic loss and neurodegeneration), and nuclei (DAPI, white) in the thorax and abdomen of male *Drosophila* from CS (control) and *Sk2* mutant groups under non-exercise (NE) and exercise (E) conditions at 3 and 6 weeks. Quantification of lipid object count, and area **(c, d, g, h),** as well as lipid and synapsin intensity **(e, f, I, j)** was performed in the thorax and abdomen tissues. *Sk2* mutant flies exhibited significantly higher lipid accumulation and synapsin intensity compared to controls, indicating metabolic dysregulation and synaptic impairment. Exercise significantly reduced obesity-associated lipid accumulation and synapsin intensity in the thorax, with minimal changes observed in the abdomen. All images were acquired using identical exposure and acquisition settings across all genotypes and conditions. Images shown are representative of ≥3 independent biological replicates. Quantitative analyses were performed from n = 5 flies/ genotype/ condition, with multiple sections analyzed per fly. Data are presented as mean ± SEM with individual data points. Statistical analysis was performed using two-way ANOVA with Tukey's multiple comparisons test. (ns = $p > 0.05$, * = $p < 0.05$, ** = $p < 0.01$, *** = $p < 0.0001$). All the raw data including all the statistical significance are shown in the source file.

Supplementary Fig. 2.

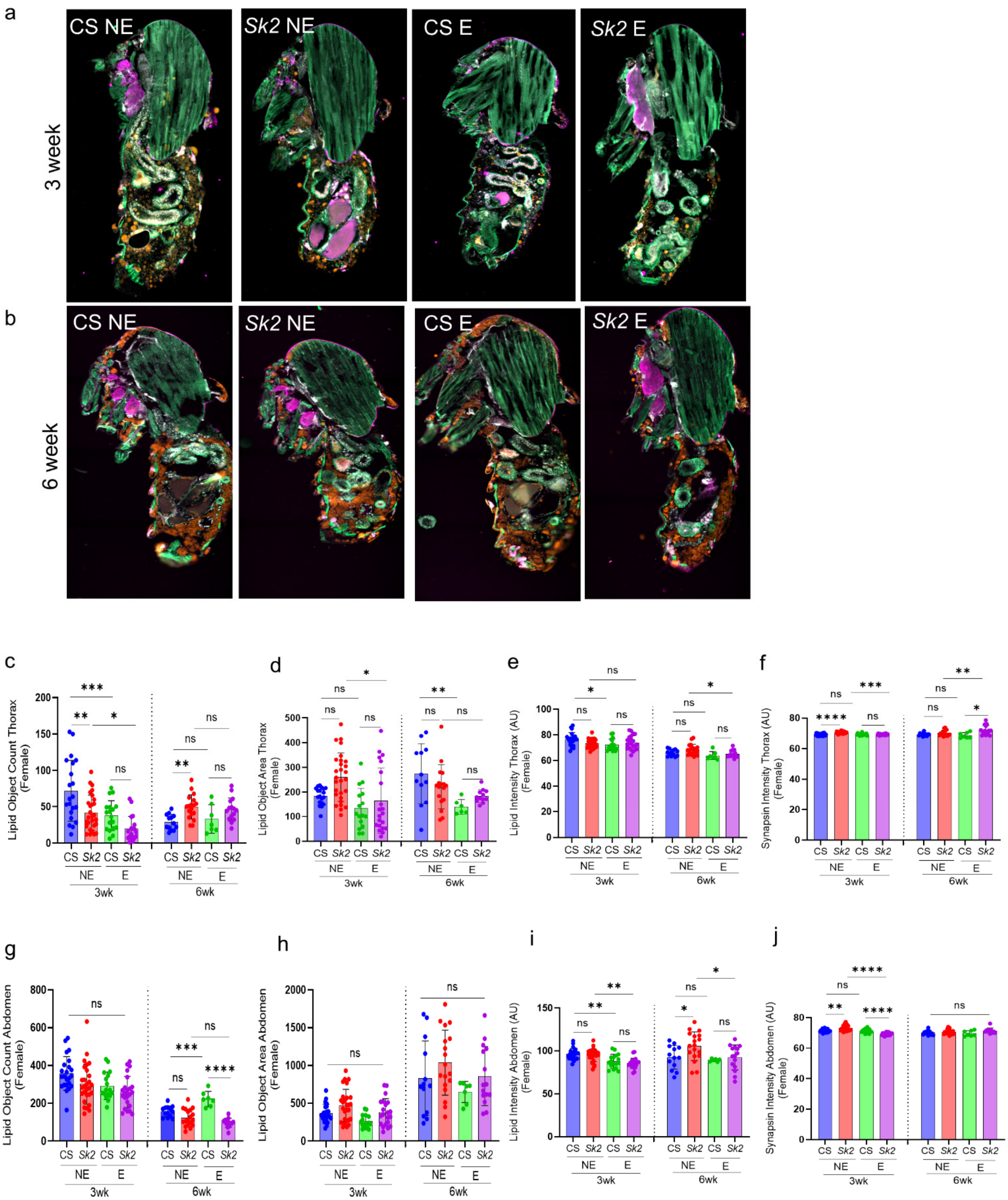

Supplementary Fig. 2. Effects of exercise on thorax and abdomen morphology in female CS and *Sk2* groups at 3 and 6 weeks.

(a, b) Representative images showing lipid accumulation (orange), phalloidin staining (green), synapsin (magenta, indicating synaptic loss and neurodegeneration), and nuclei (DAPI, white) in the thorax and abdomen female *Drosophila* from CS (control) and *Sk2* mutant groups under NE and E conditions at 3 and 6 weeks. Quantification of lipid object count and area (c, d, g, h), as well as lipid and synapsin intensity (e, f, I, j) was performed in thorax and abdomen tissues. Compared to controls, *Sk2* mutant females exhibited altered lipid accumulation and increased synapsin intensity, indicating metabolic dysregulation and synaptic alterations. Exercise reduced lipid accumulation in the thorax, with limited changes observed in the abdomen, suggesting region-specific responsiveness. All images were acquired using identical exposure and acquisition settings across all genotypes and conditions. Images shown are representative of ≥3 independent biological replicates. Data are presented as mean ± SEM with individual data points. Statistical analysis was performed using two-way ANOVA with Tukey's multiple comparisons test. (ns = $p > 0.05$, * = $p < 0.05$, ** = $p < 0.01$, *** = $p < 0.0001$). All the raw data including all the statistical significance are shown in the source file.

Supplementary Fig. 3.

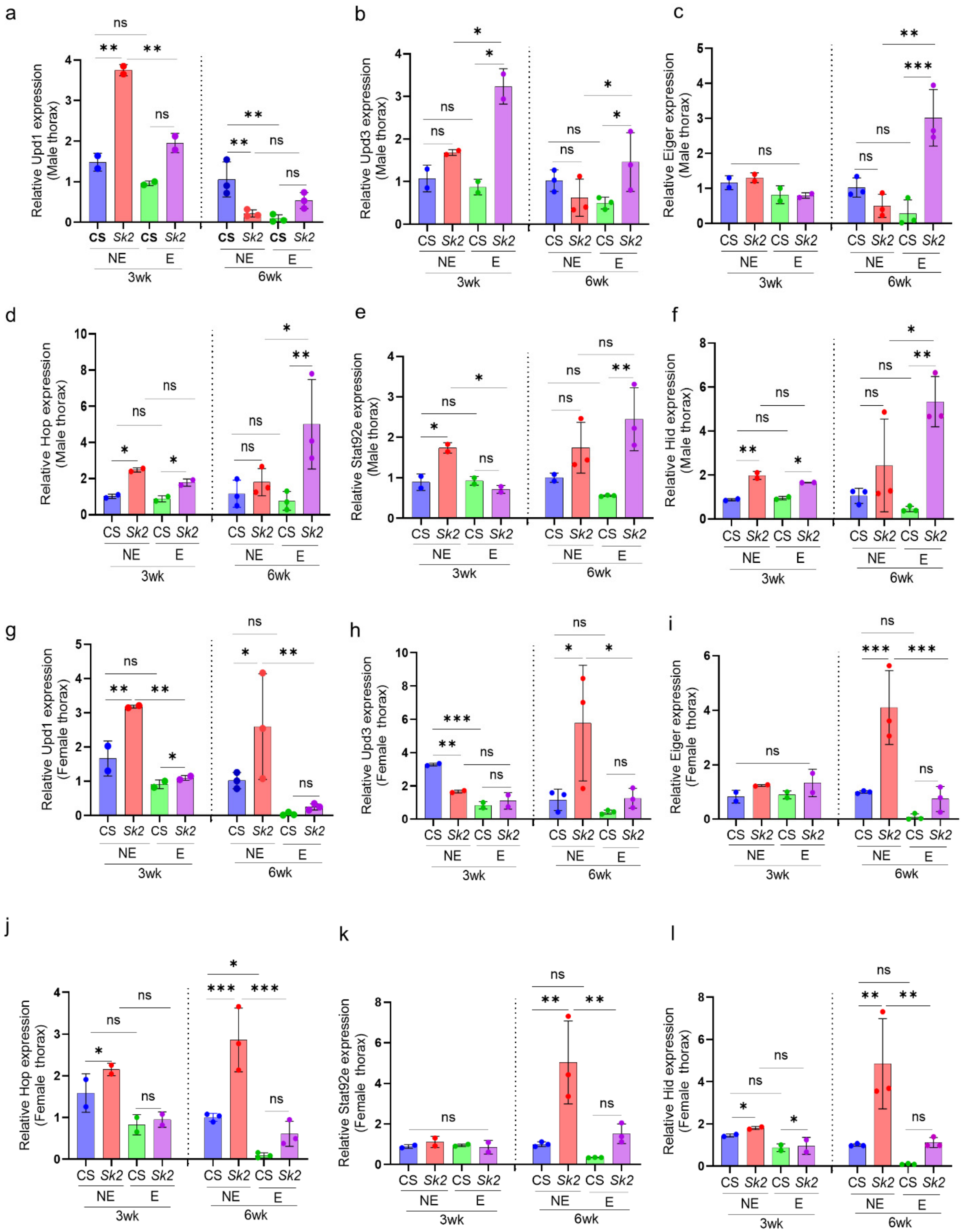

Supplementary Fig. 3. Effects of exercise on cytokine, signaling, and apoptosis-related gene expression in male and female thorax in CS and *Sk2* groups at 3 and 6 weeks. Quantitative PCR analysis of male thorax (a-f) revealed elevated expression of inflammatory cytokines (upd1, and upd3) and their signaling components (Hop, Stat), along with the pro-inflammatory marker Eiger in *Sk2* mutant brains at 3 and 6 weeks. Exercise strongly downregulated upd1, upd3, Hop, and Stat in *Sk2* mutants. Eiger expressions showed minimal change under NE conditions but increased with E at 6 weeks, suggesting a context-dependent regulatory response. Expression of the pro-apoptotic gene Hid was significantly reduced following exercise, particularly at 6 weeks, indicating suppression of apoptotic signaling. In female thorax (g–l), *Sk2* mutants similarly exhibited elevated expression of inflammatory cytokines, JAK/STAT components, and apoptotic markers compared to controls. Exercise significantly downregulated these genes, with a more pronounced effect observed at 6 weeks. Gene expression levels in CS flies remained largely unchanged with exercise. Data are presented as mean ± SEM. Statistical analysis was performed using two-way ANOVA with Tukey's multiple comparisons test (ns = $p > 0.05$, * = $p < 0.05$, ** = $p < 0.01$, *** = $p < 0.0001$). qPCR analysis was performed using n = 3 biological replicates, each consisting of pooled samples from 8–10 flies per replicate. All the raw data including all the statistical significance are shown in the source file.

Supplementary Fig. 4.

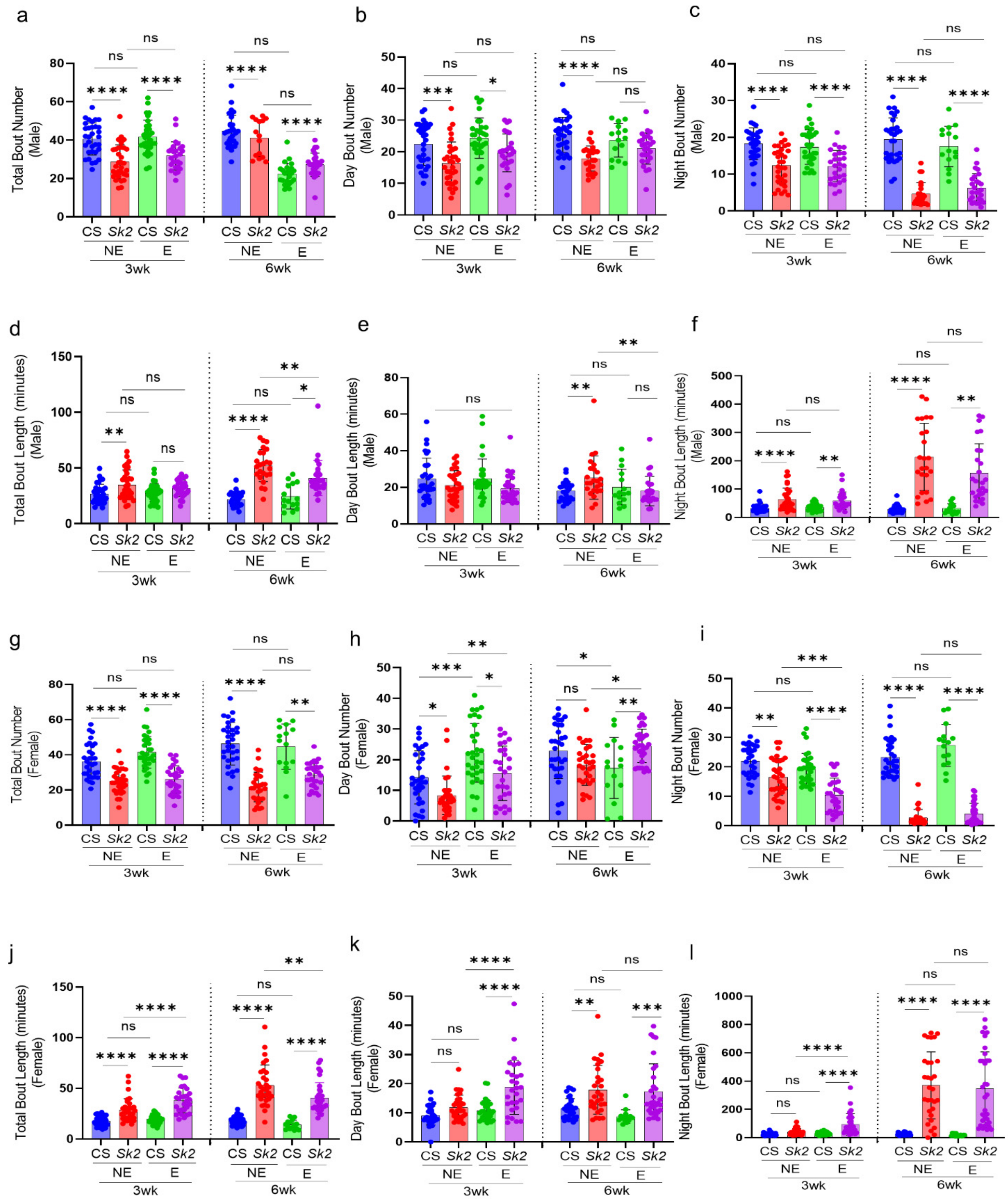

Supplementary Fig. 4. Effects of exercise on sleep bouts and bout length in CS and *Sk2* male and female *Drosophila* at 3 and 6 weeks. Obesity alters sleep architecture, including both bout length and bout number in both male **(a-f)** and female **(g-l)** flies. *Sk2* mutant flies exhibited increased total and nighttime bout lengths, along with reduced bout numbers compared to CS controls at both 3 and 6 weeks, indicating fragmented sleep patterns. These effects were more pronounced in females. Under NE conditions, *Sk2* mutants displayed significantly longer bout lengths and fewer sleep bouts during both day and night. Exercise partially rescued these alterations, resulting in improvements in total, daytime, and nighttime bout lengths, as well as increased bout numbers. These exercise-induced effects were more prominent in females. Data are presented as mean ± SEM. Statistical analysis was performed using two-way ANOVA followed by Tukey's multiple comparisons test (n = 32 flies/ genotype/ condition). Significance levels: ns = $p > 0.05$, * = $p < 0.05$, ** = $p < 0.01$, *** = $p < 0.0001$. All the raw data including all the statistical significance are shown in the source file.

Supplementary Fig. 5.

Assay/Parameter	Biological Process Evaluated	3-Week Male	3-Week Female	6-Week Male	6-Week Female	Effect of Exercise
Lipid Accumulation	Obesity phenotype	↓	↓	↓↓	↓↓	Improved
Neuroinflammation Markers	Inflammatory status	↓	↓	↓↓	↓↓	Reduced inflammation
JAK/STAT Signaling	Inflammatory pathway activation	↓	↓	↓↓	↓↓	Suppressed pathway
Learning & Memory	Cognitive function	↑	↑	↑↑	↑↑	Improved cognition
Total Sleep Duration	Sleep regulation	Improved	Improved	Improved	Improved	Beneficial
Day Sleep Duration	Circadian behavior	Improved	Improved	Improved	Improved	Beneficial
Night Sleep Duration	Circadian behavior	Improved	Improved	Improved	Improved	Beneficial
Total Sleep Fragmentation	Sleep quality	↓	↓↓	↓	↓	Reduced fragmentation
Overall Phenotype	Integrated outcome	Improved	Improved	Strongly improved	Strongly improved	Protective

Supplementary Fig. 5. Summary of the effects of exercise on metabolic, inflammatory, cognitive, and sleep-related parameters in male and female subjects at 3 and 6 weeks. Exercise reduced lipid accumulation, neuroinflammatory markers, and JAK/STAT signaling, with stronger effects observed at 6 weeks. Improvements were also observed in learning and memory, total sleep duration, day/night sleep behavior, and sleep fragmentation. Overall, exercise produced a protective phenotype, with more pronounced benefits after prolonged intervention. Arrows indicate the direction and magnitude of change (↑ increase, ↓ decrease).
